# Adherence to the healthy eating index-2010 and alternative healthy eating index-2010 in relation to metabolic syndrome among African Americans in the Jackson heart study

**DOI:** 10.1017/S1368980024000016

**Published:** 2024-02-16

**Authors:** Nicole K Reeder, Jennifer C Reneker, Bettina M Beech, Marino A Bruce, Elizabeth Heitman, Keith C Norris, Sameera A Talegawkar, Roland J Thorpe

**Affiliations:** 1 Department of Food Science, Nutrition, and Health Promotion, Mississippi State University, Mississippi State, MS, USA; 2 Department of Population Health Sciences, University of Mississippi Medical Center, Jackson, MS, USA; 3 UH Population Health, University of Houston, Houston, TX, USA; 4 Department of Behavioral and Social Sciences, University of Houston, Tilman J. Fertitta Family College of Medicine, Houston, TX, USA; 5 Program in Ethics in Science and Medicine, University of Texas Southwestern Medical Center, Dallas, TX, USA; 6 Department of Medicine, Division of General Internal Medicine and Health Services Research, David Geffen School of Medicine, University of California Los Angeles, Los Angeles, CA, USA; 7 Department of Exercise and Nutrition Sciences, Milken Institute School of Public Health, The George Washington University, Washington, DC, USA; 8 Hopkins Center for Health Disparities Solutions, Johns Hopkins School of Public Health, 624 N. Broadway, Ste 708, Baltimore, MD, USA

**Keywords:** Healthy Eating Index, Alternative Healthy Eating Index, Metabolic syndrome, Dietary quality, African Americans, Jackson Heart study

## Abstract

**Objective:**

The primary objective of this study was to determine whether Healthy Eating Index (HEI) and Alternative Healthy Eating Index (AHEI) scores were associated with incident metabolic syndrome.

**Design:**

This study is a secondary analysis of data from the Jackson Heart Study. HEI and AHEI scores were divided into quintiles and Cox proportional hazards regression models were analysed for 1864 African American adults free from metabolic syndrome at Exam 1 to examine the incidence of metabolic syndrome by quintile of dietary quality score.

**Setting:**

Hinds, Madison and Rankin counties, Mississippi, USA.

**Participants:**

African American adults, ages 21–94 years, 60·9 % female.

**Results:**

Over a mean follow-up time of 6·7 years, we observed 932 incident cases of metabolic syndrome. After adjusting for multiple covariates, a higher HEI score at Exam 1 was not associated with the risk of incident metabolic syndrome, except when looking at the trend analysis for the subgroup of adults with two metabolic syndrome components at Exam 1 (*P*_-trend_ = 0·03). A higher AHEI score at Exam 1 was associated with the risk of incident metabolic syndrome (hazard ratio for those in the highest quintile compared to the lowest: 0·80 (95 % CI: 0·65, 0·99), *P*_-trend_ = 0·03).

**Conclusion:**

These findings suggest that a dietary pattern that scores higher on the AHEI may help reduce the risk of metabolic syndrome, even for adults who already have two of the minimum of three components required for a diagnosis of metabolic syndrome.

Metabolic syndrome is characterised by a cluster of related traits, including abdominal obesity, insulin resistance, hypertension and dyslipidaemia^(1)^. The presence of these traits is concerning because, in combination, they are associated with an increased risk of developing chronic conditions such as CVD and type 2 diabetes^(1)^. The latest available data show that rates of metabolic syndrome have risen in the United States: between 1988 and 2012, the prevalence of metabolic syndrome among adults rose by approximately 35 %^(2)^. Rates of metabolic syndrome have historically been slightly lower for the African American population compared to the White population; however, this trend appears to be changing. Between 1988 and 2012, the prevalence of metabolic syndrome rose 55 % for African American men, which was more than for any other race-ethnicity-gender group in the United States^(2)^. African American women also saw a 41 % increase in metabolic syndrome prevalence during this same time period, highlighting the increased burden of metabolic syndrome for the African American population^(2)^. Furthermore, the prevalence of conditions related to metabolic syndrome, such as hypertension and type 2 diabetes, is also higher in the African American population^(3,4)^.

Modifiable lifestyle factors such as unhealthy diet, physical inactivity and cigarette smoking can increase the risk of developing metabolic syndrome^(5)^. Diet may influence the risk of metabolic syndrome either through one’s overall dietary pattern, intake of specific foods or intake of specific nutrients that may increase or decrease the risk of chronic disease^(6–12)^. One way to measure the degree of healthy dietary patterns in population-based studies is through dietary quality indices such as the Healthy Eating Index (HEI) or Alternative Healthy Eating Index (AHEI). As measured by these indices, a higher level of dietary quality has been associated with a reduced risk of chronic diseases in many large observational studies. Higher scores on the HEI, which reflects greater alignment with the Dietary Guidelines for Americans, have been associated with a lower risk of CVD, stroke, CVD mortality, cancer mortality and all-cause mortality^(13–16)^. Similarly, higher scores on the AHEI-2010, which measures intake of specific foods and nutrients associated with a reduced risk of chronic disease, have been associated with a lower risk of CVD, CVD mortality, colorectal cancer, type 2 diabetes, chronic kidney disease and excessive weight gain^(13,14,16–20)^. There is limited evidence, however, regarding whether HEI-2010 and AHEI-2010 scores are associated with incident metabolic syndrome among adults in the United States. Since metabolic syndrome and its individual components are risk factors for developing chronic conditions in the future, their association with dietary quality should be examined.

African Americans have often been underrepresented in large, population-based studies that have assessed dietary quality and risk of chronic disease, and few studies have examined dietary quality and chronic disease in an exclusively African American cohort^(18,21,22)^. Because several studies that have examined dietary quality among the African American population generally concur that dietary quality is lower among African Americans compared to White Americans^(23–28)^, understanding the relation between metabolic syndrome and dietary quality in this population is warranted. Some studies have suggested that African American adults living in the South are more likely to follow a Southern dietary pattern highlighted by a greater consumption of red and processed meats, organ meats, fried foods and sugar-sweetened beverages, which is also associated with a greater risk of CVD^(29–31)^.

Furthermore, among participants in the Black Women’s Health Study, dietary quality as measured by the Dietary Approaches to Stop Hypertension dietary index was inversely associated with mortality for African American women^(32)^, and among African American participants in the Multiethnic Cohort, AHEI-2010 scores were inversely associated with the risk of type 2 diabetes for men, but not women^(22)^. Whether similar findings would be observed for other dietary indices and other health outcomes in different African American cohorts remains unknown. Looking at the incidence of intermediate-risk conditions such as metabolic syndrome also holds important possible relevance for further disease prevention efforts. Therefore, the objective of this study was to assess dietary quality in relation to incident metabolic syndrome among African American participants in the Jackson Heart Study (JHS), using the HEI and the AHEI. The HEI was chosen because it reflects the Dietary Guidelines for Americans, which is the dietary pattern that Americans are most often encouraged to follow. The AHEI was also included because it is a modification of the original HEI that focuses primarily on foods and nutrients associated with the risk of chronic disease.

## Methods

### Study population

This study is a secondary analysis of data from the JHS, a large, prospective cohort study of CVD among African Americans residing in the metropolitan Jackson, Mississippi area (Hinds, Madison and Rankin counties)^(33,34)^. Exam 1 (baseline) data were collected between 2000 and 2004 from 5,306 adults, ages 21–94 years of age. Two subsequent follow-up visits have been completed since Exam 1, Exam 2 between 2005 and 2008 and Exam 3 between 2009 and 2013. Data were collected on sociodemographic, behavioural and biological risk factors for CVD, including diet. All JHS participants provided written informed consent prior to beginning the study, and the study protocol was approved by the Institutional Review Boards at the University of Mississippi Medical Center, Jackson State University and Tougaloo College.

**Fig. 1:**
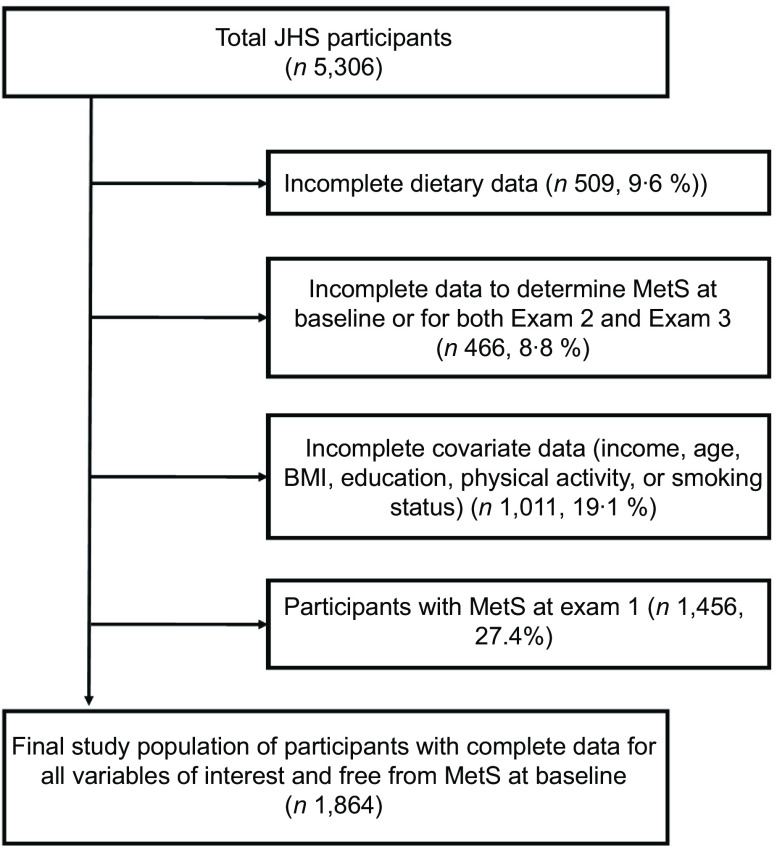
Flowchart showing the determination of the final study population

The present study included JHS participants with food frequency questionnaire data who were free of metabolic syndrome at Exam 1. Of the 5,306 JHS participants, 3,442 were excluded from analysis (Fig. 1). Participants were excluded for having an incomplete food frequency questionnaire (defined as having implausible energy intakes of <600 or >4,800 kcal/d or missing dietary data, *n* 509), incomplete data to determine MetS at Exam 1 or for both Exam 1 and Exam 2 (*n* 466), incomplete covariate data (*n* 1,011) or for having MetS at Exam 1 (*n* 1,456).

### Measures

#### Healthy Eating Index-2010 and Alternative Healthy Eating Index-2010 Scores

Dietary intake was assessed at Exam 1 using the 158-item Delta Nutrition Intervention Research Initiative JHS food frequency questionnaire (Delta NIRI JHS FFQ), which is a region-specific food frequency questionnaire validated for use with JHS participants^(35)^. The Delta NIRI JHS FFQ was administered in person by trained interviewers during Exam 1 visits. Responses to the Delta NIRI JHS FFQ were analysed using the Nutrition Data System for Research software at the Nutrition Coordinating Center (University of Minnesota, Minneapolis, MN). HEI-2010 and AHEI-2010 scores were derived from nutrient and food intake estimates of the Delta NIRI JHS FFQ.

The HEI-2010 has been validated as a means to measure dietary quality of Americans ages 2 and up, defined as degree of adherence to the *2010–2015 Dietary Guidelines for Americans* ^(36,37)^. The HEI-2010 consists of 12 food components. Nine are *adequacy* components (total fruit, whole fruit, total vegetables, greens and beans, whole grains, dairy, total protein foods, seafood and plant proteins, and fatty acids) and three are *moderation* components (refined grains, sodium and empty calories). For adequacy components, higher scores reflect a higher intake, and for moderation components, higher scores reflect a lower intake. Each component has a minimum possible score of 0 and a maximum possible score of 5, 10 or 20 points. Intake of each component is adjusted for total energy intake (intake per 1,000 kcal), and intermediate intakes of foods are scored proportionally between 0 to 5 and 0 to 10 or 20 points. Total scores for the HEI-2010 range from 0 to 100 possible points.

The AHEI is similar to the HEI but was designed with the goal of identifying and assessing intake of specific foods and nutrients that are associated with the risk of chronic disease^(18,21)^. For the AHEI-2010, 11 components are considered: total vegetables, total fruits, whole grains, sugar-sweetened beverages, nuts and legumes, red/processed meat, trans fat, long chain fatty acids, PUFA, sodium and alcohol. Each component ranges from 0-10 points, with 0 points awarded for no intake of that item and 10 points awarded for meeting or exceeding the standard for intake for that food component. Intermediate intakes of foods are scored proportionally between 0 and 10 points. Total scores for the AHEI-2010 can range from 0 to 110 possible points.

#### Metabolic syndrome

The primary outcome variable of interest in this study was incident metabolic syndrome, which is defined by the National Cholesterol Education Program Adult Treatment Panel III criteria^(38)^. Based on this definition, participants were considered to have metabolic syndrome if they met at least 3 of the 5 following conditions: elevated waist circumference (≥102 cm for males and ≥88 cm for females), elevated triglycerides (≥150 mg/dL or on drug treatment for elevated triglycerides), reduced HDL-C (<40 mg/dL for men and <50 mg/dL for women, or on drug treatment for reduced HDL-C), elevated blood pressure (systolic blood pressure ≥130 mmHg or diastolic blood pressure ≥85 mmHg or on anti-hypertensive drug treatment) and elevated fasting glucose (≥100 mg/dL, or on drug treatment for elevated glucose). Incident metabolic syndrome was considered present when a participant who had been free of metabolic syndrome at Exam 1 had at least three of the five components of metabolic syndrome present based on clinical measurements obtained at Exam 2 or Exam 3.

#### Covariates

Covariates were selected a priori based on classic confounders of diet-disease relationships and known non-dietary risk factors for MetS^(5)^. Covariates considered included age (in years), sex, BMI, family income level (poor, lower-middle, upper-middle and affluent), education level (< high school, high school graduate/GED, some college or greater), physical activity (as a sum of three index scores – active living, home life and sports) and smoking status (current smoker, past smoker and never smoker).

### Statistical analysis

For statistical analyses, HEI and AHEI scores were first divided into quintiles, in line with prior studies^(13,16,18)^. Descriptive statistics were calculated to summarise the independent variables and covariates, and independent samples *t*-tests or chi-square tests were used to compare Exam 1 characteristics between those in the highest and lowest quintiles for dietary quality. To calculate whether HEI and AHEI scores were associated with incident metabolic syndrome, Cox proportional hazards regression models were used to calculate hazard ratios and 95 % CI for risk of metabolic syndrome by quintiles of each dietary score, using the lowest quintile (representing the poorest quality diet) as the reference group. The event date was defined as years to the first follow-up exam at which a participant met the minimum criteria for metabolic syndrome. Participants who did not develop metabolic syndrome were censored at the last exam they attended. Schoenfeld residuals were examined to assess the proportional hazard assumption^(39,40)^. Different models were developed to assess the relationship between dietary quality and metabolic syndrome. Model 1 adjusted for age and sex and Model 2 adjusted for all additional covariates (income, education, physical activity and smoking status). We additionally tested for interaction between HEI and AHEI scores and sex, and HEI and AHEI scores and age, by means of interaction terms in the fully adjusted model. *P*-values for the trend analysis were determined by treating the HEI and AHEI quintile categories as ordinal variables. All tests were two-sided, and p-values less than 0·05 were considered statistically significant. All analyses were conducted using SAS version 9·4.

## Results

The final study population (*n* 1,864) had a mean age ± sd of 51·6 ± 12·5 years and was 60·9 % female (Table 1). Compared to the participants not in the analysis, those who were included were younger (*P* < 0·0001), had higher physical activity scores (*P* < 0·0001), lower HEI scores (*P* = 0·0014), were of a higher income level and higher education level and were less likely to be past or current smokers (*P* < 0·001 for each). There were no significant differences in sex distribution or AHEI scores between JHS participants included and not included in this analysis.

**Table 1:** Descriptive characteristics of Jackson Heart Study participants free of metabolic syndrome at Exam 1 by quintile of dietary quality score

	Healthy Eating Index-2010 quintile						Alternative Healthy Eating Index-2010 quintile					
Characteristic	1	SD or %	1	SD or %	3	SD or %	5	SD or %	3	SD or %	5	SD or %
Dietary quality Score^*^	47·9		40·6		51·8		64·6		60·3		75·0	
*n*	372		372		373		373		372		373	
Age (years), mean ± *sd*	49·3	12·9	48·5	12·8	51·6	6·7	55·3	7·3	50·8	11·7	56·2	11·3
Male, *n (%)*	164	44·1	147	39·5	144	38·6	142	38·1	150	40·3	119	31·9
BMI, mean ± *sd*	29·7	8·0	30·4	8·0	30·2	7·0	29·3	5·8	30·4	7·2	29·8	6·4
Income, *n (%)*												
Poor	58	15·6	62	16·7	35	9·4	34	9·1	39	10·5	34	9·1
Lower-middle	118	31·7	98	26·3	97	26·0	50	13·4	64	17·2	67	18·0
Upper-middle	105	28·2	118	31·7	125	33·5	115	30·8	138	37·1	104	27·9
Affluent	91	24·5	94	25·3	116	31·1	174	46·7	131	35·2	168	45·0
Education, *n (%)*												
<High school	70	18·8	62	16·7	46	12·3	36	9·7	36	9·7	39	10·5
High school graduate/GED	91	24·5	80	21·5	67	18·0	56	15·0	60	16·1	59	15·8
Attended vocational or trade school or college	211	56·7	230	61·8	260	69·7	281	75·3	276	74·2	275	73·7
Smoking status, *n (%)*												
Current smoker	69	18·6	44	11·8	47	12·6	39	10·5	48	12·9	20	5·4
Past smoker	63	16·9	64	17·2	45	12·1	73	19·6	67	18·0	61	16·4
Never smoker	240	64·5	264	71·0	281	75·3	261	70·0	257	69·1	292	78·3
Total physical activity score, mean ± *sd*	6·5	1·9	6·4	1·9	6·7	2·0	7·3	2·1	6·9	2·0	7·3	2·2
MetS components^†^, *n (%)*												
Waist circumference	165	44·4	184	49·5	182	48·8	174	46·7	179	48·1	179	48·0
Blood pressure	146	39·3	149	40·1	170	45·6	183	49·1	171	46·0	198	53·1
HDL cholesterol	104	28·0	116	31·2	91	24·4	66	17·7	86	23·1	76	20·4
Fasting glucose	29	7·8	23	6·2	31	8·3	38	10·2	24	6·5	34	9·1
Triglycerides	21	5·7	14	3·8	21	5·6	11	3·0	22	5·9	11	3·0

* Median score for quintile.

† MetS, metabolic syndrome. Number of participants meeting each of the individual criteria at Exam 1.

The mean HEI-2010 score for all participants free of metabolic syndrome at Exam 1 was 60·8 ± 10·5, and the mean AHEI-2010 score for participants was 52·4 ± 9·3. Most participants met at least one criterion for metabolic syndrome, with 46·3 % (*n* 863) meeting two criteria and 36·4 % (*n* 679) meeting one criterion. Only 17·3 % (*n* 322) met none of the criteria for metabolic syndrome at Exam 1. Looking at individual metabolic syndrome criteria met by participants free of metabolic syndrome at Exam 1, 47·5 % had an elevated waist circumference, 44·7 % had elevated blood pressure or were on an anti-hypertensive medication, 24·5 % had a low HDL level or were taking a statin medication, 7·9 % had an elevated fasting plasma glucose level or were on a hypoglycaemic medication, and 4·4 % had an elevated triglyceride level.

Participants in the highest quintile for HEI score were more likely to be older, female, non-smoking, and have a higher income level, higher attained education level, higher physical activity score and elevated blood pressure compared to participants in lower quintiles (Table 1). Similar findings were observed for AHEI scores, with the exception of sex and smoking status appearing more similar across AHEI quintiles.

### Healthy Eating Index-2010, Alternative Healthy Eating Index-2010 and incident metabolic syndrome

Over a mean follow-up time of 6·7 ± 1·8 years, 932 of the 1,864 participants (50·0 %) developed metabolic syndrome by either their Exam 2 or Exam 3 visit. By quintile of HEI-2010 score, incidence rates per 1,000 person-years ranged from 71·0 (Quintile 2) to 80·5 (Quintile 5). The crude model showed no significant association between HEI quintile and incident metabolic syndrome (Table 2). There remained no significance after adjusting for age, sex and BMI (Model 1) and after further adjusting for income level, education level, physical activity and smoking status (Model 2). There were no significant interaction effects between HEI-2010 quintiles and sex or HEI-2010 quintiles and age (*P* > 0·05 for all).

**Table 2:** Risk of metabolic syndrome by quintile of Healthy Eating Index (HEI-2010) score among Jackson Heart Study participants (Exam 1, 2000–2004)

	Quintile 1	Quintile 2	95 % CI	Quintile 3	95 % CI	Quintile 4	95 % CI	Quintile 5	95 % CI	*P*-trend
Cases^*^	195	176		185		181		195		–
Cases/person-years	195/2558·1	176/2478·4		185/2487·7		181/2470·5		195/2422·8		–
Incidence rate/1000 person-years	76·2	71·0		74·4		73·3		80·5		–
HR^†^										
Crude model	Reference	0·98	0·80, 1·21	1·05	0·86, 1·28	1·03	0·84, 1·27	1·17	0·96, 1·43	0·11
Model 1	Reference	0·99	0·81, 1·23	1·02	0·83, 1·24	0·98	0·80, 1·20	0·99	0·81, 1·21	0·87
Model 2	Reference	0·99	0·80, 1·21	1·00	0·81, 1·22	0·97	0·79, 1·20	0·99	0·80, 1·23	0·90

* Values are hazard ratios (95 % CI). Model 1 is adjusted for age and sex. Model 2 is adjusted for age, sex, income, education, physical activity and smoking status. Age and physical activity scores were treated as continuous variables. The remaining covariates were categorical with sex=female, income=poor, education= < high school and smoking status current smoker as the reference categories.

† A case is defined as a person without metabolic syndrome at Exam 1 who was found to have metabolic syndrome at either Exam 2 or Exam 3.

By quintile of AHEI-2010 score, incidence per 1,000 person-years ranged from 72·8 (Quintile 2) to 79·3 (Quintile 1). There was no statistically significant association between AHEI-2010 quintiles and incident metabolic syndrome in the crude model or in Model 1 (Table 3). In the fully adjusted model, Quintile 5 was significantly associated with a lower risk of incident metabolic syndrome (HR 0·80, CI: 0·65, 0·99), and there was a significant association between AHEI quintiles and incident MetS in the trend analysis (*P* = 0·03). Finally, there were no significant interaction effects between AHEI quintiles and age or sex (*P* > 0·05 for all).

**Table 3:** Risk of metabolic syndrome by quintile of Alternative Healthy Eating Index (AHEI-2010) score among Jackson Heart Study participants (Exam 1, 2000–2004)

	Quintile 1	Quintile 2	95 % CI	Quintile 3	95 % CI	Quintile 4	95 % CI	Quintile 5	95 % CI	***P*** **-trend**
Cases	198	180		186		187		181		–
Cases/person-years	198/2496·3	180/2473·3		186/2484·5		187/2494·2		181/2469·2		
Incidence rate/1000 person-years	79·3	72·8		74·9		75·0		73·3		
HR^†^										
Crude model	Reference	0·94	0·77, 1·16	0·97	0·80, 1·19	0·96	0·79, 1·17	0·97	0·79, 1·19	0·83
Model 1	Reference	0·93	0·76, 1·14	0·93	0·76, 1·14	0·87	0·71, 1·07	0·84	0·68, 1·03	0·07
Model 2	Reference	0·92	0·75, 1·13	0·90	0·74, 1·10	0·83	0·68, 1·02	0·80^*^	0·65, 0·99	0·03

* *P* < 0·05.

† Values are hazard ratios (95 % CI). Model 1 is adjusted for age and sex. Model 2 is adjusted for age, sex, income, education, physical activity and smoking status. Age and physical activity scores were treated as continuous variables. The remaining covariates were categorical with sex=female, income=poor, education= < high school and smoking status current smoker as the reference categories.

Additional analyses stratified by the number of metabolic syndrome criteria participants met at Exam 1 were conducted (Tables 4 and 5). Nearly half of the participants without metabolic syndrome at Exam 1 already met two of the three criteria required for the diagnosis (*n* 863, 46·3 %). Incidence rates for those with two criteria were higher for those with lower dietary quality, ranging from 101·5 cases per 1000 person-years (HEI Quintile 4) to 123·8 cases per 1000 person-years (HEI Quintile 1), and for the AHEI, from 101·6 cases per 1000 person-years (Quintile 5) to 117·1 cases per 1000 person-years (Quintile 1). Only 322 (17·3 %) of participants met zero criteria for metabolic syndrome at Exam 1. For adults with two MetS criteria at Exam 1, a trend analysis showed a significant decrease in MetS incidence with increasing HEI and AHEI scores (Tables 4 and 5). For adults with one MetS criteria at Exam 1, there was a significant trend in MetS incidence with higher HEI-2010 scores suggesting a higher incidence of MetS, but this trend lost significance after adjusting for various covariates in Model 2 and Model 3 (Table 4). Finally, for adults with zero criteria for MetS at Exam 1, results from Cox proportional hazards regression models showed no association between HEI or AHEI quintiles and MetS incidence (Tables 4 and 5).

**Table 4:** Risk of metabolic syndrome by quintile of Healthy Eating Index-2010, stratified by number of metabolic syndrome criteria present at Exam 1(2000–2004)

	Quintile 1	Quintile 2	95 % CI	Quintile 3	95 % CI	Quintile 4	95 % CI	Quintile 5	95 % CI	***P*** **-trend**
Two MetS criteria (*n* 863)										
Cases	124	118		116		116		115		–
Incidence rate/1000 person-years	123·8	118·2		109·0		101·5		104·4		–
HR^*^										
Crude model	Reference	1·04	0·80, 1·34	0·93	0·72, 1·20	0·88	0·68, 1·13	0·93	0·72, 1·20	0·29
Model 1	Reference	1·05	0·82, 1·36	0·92	0·71, 1·19	0·84	0·65, 1·09	0·84	0·65, 1·09	0·06
Model 2	Reference	1·04	0·81, 1·35	0·87	0·67, 1·13	0·82	0·63, 1·06	0·80	0·61, 1·05	0·03
One MetS criteria (*n* 679)										
Cases	57	47		54		51		66		–
Incidence rate/1000 person-years	56·1	49·1		54·7		60·8		72·1		–
HR^*^										
Crude model	Reference	0·91	0·62, 1·34	1·01	0·69, 1·46	1·14	0·78, 1·66	1·42	0·99, 2·03	0·03
Model 1	Reference	0·91	0·61, 1·34	0·99	0·68, 1·44	1·09	0·75, 1·59	1·24	0·86, 1·78	0·16
Model 2	Reference	0·89	0·60, 1·31	1·00	0·68, 1·47	1·09	0·73, 1·62	1·23	0·84, 1·80	0·17
Zero MetS criteria (*n* 322)										
Cases	14	11		15		14		14		–
Incidence rate/1000 person-years	25·9	21·0		34·4		28·6		34·4		–
HR^*^										
Crude model	Reference	1·04	0·80, 1·34	0·93	0·72, 1·20	0·88	0·68, 1·13	0·93	0·72, 1·20	0·16
Model 1	Reference	0·92	0·41, 2·05	1·70	0·81, 3·59	1·29	0·61, 2·74	1·36	0·63, 2·94	0·27
Model 2	Reference	0·94	0·41, 2·15	1·66	0·78, 3·54	1·22	0·55, 2·74	1·39	0·62, 3·14	0·28

* Values are hazard ratios (95 % CI). Model 1 is adjusted for age and sex. Model 2 is adjusted for age, sex, income, education, physical activity and smoking status. Age and physical activity scores were treated as continuous variables. The remaining covariates were categorical with sex=female, income=poor, education= < high school and smoking status current smoker as the reference categories.

**Table 5:** Risk of metabolic syndrome by quintile of Alternative Healthy Eating Index-2010, stratified by number of metabolic syndrome criteria present at Exam 1 (2000–2004)

	Quintile 1	Quintile 2	95 % CI	Quintile 3	95 % CI	Quintile 4	95 % CI	Quintile 5	95 % CI	***P*** **-trend**
Two MetS criteria (*n* 863)										
Cases	122	120		130		109		108		–
Incidence rate/1000 person-years	117·1	113·8		115·4		106·8		101·6		–
HR^*^										
Crude model	Reference	1·05	0·81, 1·35	1·07	0·83, 1·37	0·94	0·72, 1·21	0·94	0·72, 1·22	0·42
Model 1	Reference	1·03	0·80, 1·33	1·03	0·81, 1·33	0·85	0·66, 1·11	0·86	0·66, 1·11	0·10
Model 2	Reference	0·99	0·77, 1·29	1·00	0·78, 1·29	0·82	0·63, 1·08	0·80	0·61, 1·05	0·04
One MetS criteria (*n* 679)										
Cases	64	42		45		68		56		–
Incidence rate/1000 person-years	62·0	50·1		53·2		60·9		63·5		–
HR^*^										
Crude model	Reference	0·81	0·55, 1·20	0·86	0·58, 1·25	0·98	0·69, 1·38	1·08	0·76, 1·55	0·49
Model 1	Reference	0·82	0·55, 1·20	0·81	0·55, 1·19	0·93	0·66, 1·31	0·92	0·64, 1·33	0·88
Model 2	Reference	0·80	0·53, 1·18	0·76	0·51, 1·12	0·86	0·60, 1·24	0·85	0·57, 1·25	0·53
Zero MetS criteria (*n* 322)										
Cases	12	18		11		10		17		–
Incidence rate/1000 person-years	28·4	31·0		21·5		28·1		32·4		–
HR^*^										
Crude model	Reference	1·05	0·51, 2·25	0·71	0·31, 1·6	1·04	0·44, 2·41	1·11	0·53, 2·41	0·82
Model 1	Reference	0·95	0·46, 2·03	0·71	0·31, 1·62	0·96	0·40, 2·24	0·97	0·45, 2·12	0·95
Model 2	Reference	1·11	0·52, 2·45	0·79	0·33, 1·84	0·99	0·41, 2·37	0·98	0·45, 2·22	0·85

* Values are hazard ratios (95 % CI). Model 1 is adjusted for age and sex. Model 2 is adjusted for age, sex, income, education, physical activity and smoking status. Age and physical activity scores were treated as continuous variables. The remaining covariates were categorical with sex=female, income=poor, education= < high school and smoking status current smoker as the reference categories.

## Discussion

In this study of 1,864 African American adults from the JHS, we sought to examine whether dietary quality, as measured by the HEI-2010 and AHEI-2010, is associated with the risk of developing metabolic syndrome. While prior work has established an inverse relationship between HEI and AHEI scores and outcomes such as all-cause mortality, cardiovascular mortality, and CVD^(13–16,18)^, less is known about the predictive relevance of HEI and AHEI scores in relation to metabolic syndrome or any of its individual components. We found that for the HEI, higher dietary quality at Exam 1 was not significantly associated with incident metabolic syndrome in the crude or adjusted models; however, for the AHEI, participants in the highest quintile for dietary quality had a significantly lower risk of incident MetS. Further, among adults without MetS at Exam 1, those who already had two components of MetS appeared most likely to have a decreased risk of developing MetS with increasing HEI or AHEI scores. Overall, these findings suggest that higher dietary quality, as measured by the HEI-2010 and AHEI-2010, may reduce the risk of MetS among adults at heightened risk of MetS.

Baseline (Exam 1) characteristics of the participants in this study categorised by HEI and AHEI scores were similar to those observed in other cohorts^(13–16,18,19)^. Participants with higher dietary quality scores tended to be older, female, have a higher attained education level, higher income level and higher scores for physical activity level. The mean HEI score for the participants included in this analysis (e.g. those free of metabolic syndrome at Exam 1) was 60·8, which suggests that dietary quality for the participants included in this analysis was better than the mean dietary quality score for the US population, which was 49·8 (men) and 52·7 (women) among those surveyed in the 2003–2004 NHANES^(37)^. We also found that for the HEI, a greater percentage of participants in Quintile 5 had hypertension compared to those in Quintile 1, and participants in the highest quintile for the HEI also had a greater percentage of participants with an elevated waist circumference and elevated fasting glucose levels compared to those with the lowest HEI scores. These findings run counter to expectations, in that participants who had higher HEI scores tended to have a less metabolically healthy profile, showing greater abdominal adiposity, a higher prevalence of hypertension and elevated fasting blood glucose levels.

This finding is perhaps not entirely surprising, however, as several other large studies have also observed similar counterintuitive findings with the HEI. For example, cross-sectional data from NHANES 2001–2016 found that three of the criteria for metabolic syndrome were associated with a higher HEI-2015 score: use of an anti-hypertensive medication, use of lipid-lowering medication and use of hypoglycaemic medication^(41)^. Cross-sectional data from the Framingham Heart Study cohort similarly found that participants with a higher level of adherence to the HEI-2015 were more likely to have hypertension, be using an anti-hypertensive medication and be using a lipid-lowering medication; again, key components of the metabolic syndrome^(42)^. Moreover, in the Multiethnic Cohort, participants in the highest quintile for HEI-2015 scores had a higher prevalence of diabetes compared to participants in the lowest quintile for the HEI-2015^(15)^, and in the Atherosclerosis Risk in Communities (ARIC) cohort, higher HEI-2015 and AHEI-2010 scores were associated with hypercholesterolaemia^(16)^. Inconsistencies in associations between dietary quality indices and chronic disease have also been observed in the Multiethnic Cohort when analyses are stratified by sex and race^(22)^. For example, higher scores on the HEI-2010 were associated with a reduced risk of type 2 diabetes for White women, but not for any other sex-ethnicity group, and higher scores on the AHEI-2010 were associated with a reduced risk of type 2 diabetes for African American men and White women, but not any other groups^(22)^. The link between higher HEI scores and greater use of anti-hypertensive medications, lipid-lowering medications and hypoglycaemic medications is also interesting, but may be a reflection of changes in dietary habits post-diagnosis.

While few studies have been conducted to examine HEI and metabolic syndrome among US adults, one study using data from the Framingham Heart Study Offspring Cohort found that greater adherence to the 2005 dietary guidelines for Americans was associated with decreased odds of metabolic syndrome only when participants who were being treated for any of the components of metabolic syndrome were excluded from analysis^(43)^. Overall, it seems that most previous studies that have been conducted found significant associations between the HEI and AHEI and incident CVD^(13,16,18,42)^, CVD mortality^(13,44)^ and all-cause mortality^(13,44)^, while evidence for the more intermediate outcomes of metabolic syndrome, type 2 diabetes, hypertension, dyslipidaemia and obesity is limited or inconsistent.

One possible explanation for the lack of association between HEI and incident MetS observed in this study is the age of the population being studied and the length of time they were followed, as both the incidence of chronic disease and dietary quality tend to increase with age^(27,45)^. One-third of adults ages 45–64 have two or more chronic conditions, and almost two-thirds of adults ages 65 and older have two or more chronic conditions^(45)^. Older adults ages 65+ not only tend to score better than younger adults on dietary quality indices, but actually have the highest dietary quality of all Americans^(27)^. In this study, the participants free of metabolic syndrome at Exam 1 had a mean age of 51, and over 80 % already met either one or two of the three criteria for metabolic syndrome. Older adults may become more health conscious with age in reaction to a chronic disease diagnosis, or they may become more motivated to make health behaviour changes as they age to prevent chronic disease. Adults with a chronic disease also may see a healthcare provider more frequently and consequently be exposed to more frequent health promotion messaging, leading to lifestyle changes such as a healthier diet^(46)^. This factor, coupled with the much shorter follow-up time in this study compared to other studies that found the HEI and AHEI to be associated with incident CVD and other health outcomes, may explain why the HEI did not appear to be as strongly associated with incident metabolic syndrome in this cohort. Finally, there is also the potential for selection bias as the participants included in this analysis were already eating better than the average American adult based on their HEI scores. Since 44 % of participants with complete data were removed from the analysis for already having metabolic syndrome at Exam 1, it is also possible that we had selected a group more resistant to developing metabolic syndrome. If so, there may be limited association with diet quality in this group.

There were several strengths to this study. First, this study examined indices of dietary quality in an exclusively African American population. Many prior studies looking at dietary quality in relation to health outcomes have relied on cohorts such as the Nurses’ Health Study and Health Professional’s Follow-Up Study^(14,17,18,20,21)^, which are fairly homogenous cohorts of mostly White health professionals. Even in studies conducted with the ARIC cohort^(13,16,19)^, which recruited exclusively African Americans at their Jackson, Mississippi study site, the question remains whether analyses conducted by race reflect differences associated with race or whether observed differences are related to geographic location. Another strength of this study is the use of a regionally validated food frequency questionnaire designed specifically for the Southern US population and administered by trained interviewers. However, with any food frequency questionnaire, there are also limitations associated with self-reported dietary information, such as the tendency for recall bias. The food frequency questionnaire was also administered at Exam 1 only, and thus, any dietary changes over the follow-up period are not accounted for. An additional limitation of this study is that we did not look at individual component scores of the HEI and AHEI, which might have offered further insight into the role of diet on the incidence of metabolic syndrome. Individual component scores of the HEI and AHEI were not analysed in this study as we aimed to look holistically at how overall dietary pattern affects the risk of MetS. Further, the HEI is regularly updated as the Dietary Guidelines for Americans are revised in accordance with the latest evidence available. An HEI-2015 index has been released, and an HEI-2020 index is forthcoming^(47)^. Future studies may consider how adherence to the most recent Dietary Guidelines for Americans affects the risk of developing metabolic syndrome. In addition, adherence to diets such as the Dietary Approaches to Stop Hypertension Diet or the Mediterranean Diet should be evaluated as it relates to incident metabolic syndrome. While this study accounted for many potential confounding variables, the observational nature of the JHS leaves the potential for residual confounding. Variables that are not accounted for here include changes in diet over time, family history of chronic disease, date of diagnosis of components of MetS participants already had at Exam 1, alcohol intake and chronic stress. Other limitations to consider are that, despite its large population at Exam 1, this study may have been underpowered to determine the outcomes of interest and may have selection bias present. Measuring incident MetS necessitated removing all participants who already had MetS at Exam 1 from the analysis. After first removing all participants who had incomplete or implausible dietary data, incomplete data to determine the presence of MetS at follow-up and incomplete covariate data, only 3,320 participants remained, of which, 1,456 (44 %) already had MetS. This resulted in a final study sample of *n* 1,864, which is less than half of the original JHS participants. Finally, the mean follow-up time in this study was seven years, which, coupled with the characteristics of the study population (middle-aged adults, almost half of whom already have two of the minimum of three components for metabolic syndrome), may not have been enough time to capture whether greater adherence to a healthy dietary pattern helps prevent metabolic syndrome.

In summary, for adults at the greatest risk of developing MetS (those who already have at least two components), greater adherence to the HEI-2010 and AHEI-2010 was associated with a decreased incidence of metabolic syndrome over a seven-year follow-up period in this prospective cohort of 1,865 African American adults from the JHS. The AHEI-2010 as a measure of dietary quality may be a slightly better predictor of MetS risk, as an inverse relationship was found between AHEI quintiles and incident MetS, while for the HEI, such an association was only observed among adults with two MetS components at Exam 1. Overall, these findings highlight the importance of following a healthy dietary pattern and suggest that even if a person already has multiple components of MetS, greater adherence to dietary guidance may be protective against the further development of metabolic comorbidities.

Further research is needed to elucidate more fully the relationship between dietary quality and metabolic syndrome and the incidence of individual components of metabolic syndrome. Future studies examining whether dietary quality is associated with incident metabolic syndrome may benefit from following a younger cohort over a longer period of time, as most middle-aged adults already have at least one chronic condition and may have already altered their eating behaviour in response. While adherence to an overall healthy dietary pattern remains an important public health recommendation for the prevention of chronic disease, more work is needed to understand the specifics of this general effect.
